# Dose relationship between oral glucocorticoids and tumor necrosis factor inhibitors and the risk of hospitalized infectious events among patients with rheumatoid arthritis

**DOI:** 10.1007/s00296-017-3679-4

**Published:** 2017-03-02

**Authors:** Jennifer Schenfeld, Jan Iles, Mona Trivedi, Neil A. Accortt

**Affiliations:** 1 DOCS Global, Inc., North Wales, PA USA; 2 Amgen Inc., One Amgen Center Drive, Thousand Oaks, CA 91320 USA

**Keywords:** Arthritis, Rheumatoid, Glucocorticoids, Infection, Hospitalization

## Abstract

The objective of this study was to evaluate the impact of oral glucocorticoid (GC) dose on rates of hospitalized infectious events (HIEs) among RA patients newly exposed to tumor necrosis factor inhibitor (TNFi) therapy. This retrospective cohort study used data from the MarketScan claims database. Incident and prevalent adult RA patients newly exposed to TNFi therapy were identified and assigned to three cohorts: no GC, low-dose GC (≤7.5 mg), and high-dose GC (>7.5 mg); patients could contribute exposure time to multiple cohorts if they changed dose or discontinued GC. The primary outcome was estimated incidence rate (IR) of HIEs per 100 patient-years of GC exposure. A total of 40,933 eligible patients were identified (mean age 53.0 years; 77.4% female). HIE risk increased with increasing GC dose: the IR [95% confidence interval (CI)] was 3.9 (3.63–4.13) for no GC; 6.4 (5.68–7.16) for low-dose GC; and 13.3 (11.9–15.5) for high-dose GC. Adjusted rate ratios (95% CI) were 1.4 (1.21–1.60) for low-dose vs no GC; 2.8 (2.32–3.34) for high-dose vs no GC, and 2.0 (1.66–2.45) for high-dose vs low-dose GC. The risk of HIEs increased with increasing age. HIE risk did not increase with longer exposure to GCs. Oral GCs, regardless of dose, significantly increased the risk of HIEs among RA patients newly initiating TNFi therapy. Steroid dosing must be considered when assessing infection risk in treatment decisions for RA patients.

## Introduction

Rheumatoid arthritis (RA) is characterized by persistent inflammation in joints that leads to damage in the surrounding cartilage and bone. Patients with RA have been shown to be at increased risk for hospitalized infectious events (HIEs) compared to the general population [1, 2], and the risk increases with more severe disease [3]. A retrospective longitudinal study showed that the rate of infections requiring medical care among patients diagnosed with RA (19.6 cases per 100 person-years) was greater than the rate for patients without an RA diagnosis (12.9 cases per 100 person-years) [1]. In a retrospective study of HIEs, the adjusted hazard ratio (HR) for infection was 2.03 [95% confidence interval (CI) 1.93–2.13] for patients with RA vs those without RA [2]. It is not clear if this increased risk is due to dysregulation of the immune system characteristic of inflammatory diseases.

Current therapies for the treatment of RA include many drugs that have immunosuppressive properties, such as tumor necrosis factor inhibitor (TNFi) medications and glucocorticoids (GCs). A prospective observational study found that patients with RA treated with a TNFi had 20% greater risk of serious infection (defined as requiring intravenous antibiotics or hospitalization or resulting in death) compared to patients with RA treated with nonbiologic disease-modifying antirheumatic drugs (DMARDs) (adjusted HR 1.2; 95% CI 1.1–1.5) [4]. Notably, serious infections were infrequent in both groups, with rates of 42 and 32 serious infections per 1000 person-years in TNFi-treated and nonbiologic DMARD-treated patients, respectively.

Studies have suggested that oral GCs further increase the risk of HIEs among patients with RA. Although definitions of low and high dose vary between studies, exposure to low-dose GCs has been shown to increase the risk of HIEs, and exposure to high-dose GCs further increased the risk of serious infection and HIEs [5–9]. The purpose of this study was to better understand the impact of concomitant use of GCs among RA patients who were newly exposed to TNFi therapy. We estimated the incidence rates (IRs) of HIEs based on exposure to TNFi medications (both collectively and individually) and to exposure to various levels of GCs. The hypothesis to be tested was that the rate of HIEs is higher among patients with RA on TNFi medications exposed to low-dose oral GCs as compared to patients on TNFi medications who are not exposed to oral GCs.

## Materials and methods

### Study design and data source

This was a retrospective cohort study that utilized the MarketScan Commercial and Medicare supplemental claims database from January 1, 2005 through June 30, 2014. The commercial database contains the inpatient, outpatient, and outpatient prescription drug experience of ~40 million employees and their dependents, who are covered under a variety of fee-for-service and managed care health plans. The Medicare database contains the healthcare experience of ~3 million retirees with Medicare supplemental insurance paid for by employers. Both databases contain detailed cost, use, and outcomes data for inpatient and outpatient healthcare services. The medical claims are linked to outpatient prescription drug claims and person-level enrollment data through the use of unique enrollee identifiers.

The index date was the first exposure to TNFi therapy (adalimumab, certolizumab pegol, etanercept, golimumab, or infliximab). The baseline period was the 6 months preceding the index date, which was used to ensure that patients were TNFi-naïve. The follow-up period began on the index date for each patient and lasted up to 2 years. Follow-up ended on the earliest date of: disenrollment from MarketScan, HIE outcome diagnosis date, discontinuation of TNFi therapy, end of study (June 30, 2014), or 2 years after index date.

TNFi exposure was based on time from index date until the patient discontinued use of TNFi medication. Patients were permitted to switch TNFi medication if there was no interruption in TNFi use without truncating the time of follow-up. Exposure to oral GCs was assessed during the follow-up period and was categorized as: no exposure, very low dose (≤5.0 mg), low dose (≤7.5 mg), high dose (>7.5 mg), and very high dose (>20 mg). A sensitivity analysis of a dose ≥10 mg based on categories of low-dose and high-dose GCs as defined by the American College of Rheumatology (ACR) [10] was also performed. All steroid claims were converted to a 5 mg prednisone equivalent dose. Patients could have multiple oral GC exposures and could contribute time to different GC exposure categories, including the ‘no exposure’ category when they were off GCs. For example, a patient could have had several claims for varying low or high GC doses over the follow-up time, as well as intervals without GC claims. Each GC exposure was then allocated to the appropriate GC dose cohort and the time that patients were not receiving any GC was allocated to the no GC cohort. Cumulative episodic exposures for each dose cohort were measured. To accommodate the time required for drug clearance, the exposure time for high-dose exposures was extended an additional 3 days. If the patient transitioned directly from a high-dose to a low-dose GC, the low-dose exposure time was delayed by 3 days and the initial 3 days after the dose decrease were attributed to the high-dose GC. Exposure to other RA medications, such as nonbiologic DMARDs, was collected only during the baseline period.

### Patients

Eligible patients were aged ≥18 years and had a confirmed diagnosis of RA [International Classification of Diseases, Ninth Revision, Clinical Modification (ICD-9-CM) code 714.0] defined by one inpatient or one outpatient diagnostic claim accompanied by ≥1 prescription for a TNFi medication within 30 days prior to and ≤365 days after an RA claim date. Patients were required to have ≥12 months of continuous enrollment with pharmacy coverage, which included the 6-month baseline period and at least 6 months of follow-up. Patients were excluded if they had a confirmed diagnosis of psoriasis, psoriatic arthritis, ankylosing spondylitis, and/or inflammatory bowel disease (other indications for TNFi medications), a confirmed diagnosis of HIV/AIDS, any malignancy (excluding nonmelanoma skin cancer), receipt of an organ transplant before index date, exposure to a TNFi medication during the baseline period, and any inpatient HIE diagnostic claim with one overnight hospitalization stay in which the HIE diagnosis was the primary or secondary reason for hospitalization during the 90 days preceding and including the index date.

### Study outcomes

The rate of incident HIEs was estimated for each oral GC exposure group, for collective TNFi exposures, and for individual TNFi medication exposures. Rate ratios comparing the incidence rates of patients with low- and high-dose GC exposures were calculated.

### Statistical considerations

Incidence rates (IRs) of HIEs and 95% CIs were calculated per 100 patient-years. Univariate and multivariable Poisson regression models were used to assess the association between TNFi exposure, oral GC exposure, demographics, baseline comorbidities, concomitant medications, and HIEs. Covariates with *P* < 0.20 in univariate models were included in the initial multivariable model. Further evaluation was conducted to produce the most parsimonious model. Baseline demographics and clinical characteristics were based on GC category on the index date; categories were mutually exclusive at baseline.

## Results

### Patients

A total of 40,933 eligible patients were identified in the database. At baseline, the distribution was as follows: 28,867 patients (70.5%) on no GC, 9011 (22.0%) on low-dose GC, and 3055 (7.5%) on high-dose GC. Within the low-dose GC cohort, a subset of 8632 were on very low-dose GC and within the high-dose GC cohort, 131 were on very high dose GC. Most patients were women (77.4%), and the mean age was 53.0 [standard deviation (SD) 12.6] years (Table 1).

**Table 1:** Baseline demographic and clinical characteristics

	No GC *N* = 28,867	Very low dose GC^a^ (≤5.0 mg) *N* = 8632	Low-dose GC (≤7.5 mg) *N* = 9011	High-dose GC (>7.5 mg) *N* = 3055	Very high dose GC^b^ (>20.0 mg) *N* = 131
Age, mean years	52.5	54.2	54.3	53.4	51.6
Age category, *n* (%)					
<65 years	25,606 (88.7)	7345 (85.1)	7642 (84.8)	2662 (87.1)	117 (89.3)
≥65 years	3261 (11.3)	1287 (14.9)	1369 (15.2)	393 (12.9)	14 (10.7)
Sex, *n* female (%)	22,818 (79.0)	6459 (74.8)	6731 (74.7)	2130 (69.7)	91 (69.5)
Select comorbidities, *n* (%)					
Hypertension	2,722 (9.4)	938 (10.9)	981 (10.9)	370 (12.1)	14 (10.7)
Diabetes	1891 (6.6)	475 (5.5)	496 (5.5)	204 (6.7)	9 (6.9)
Congestive heart failure	574 (2.0)	223 (2.6)	241 (2.7)	105 (3.4)	5 (3.8)
Asthma	435 (1.5)	113 (1.3)	122 (1.4)	71 (2.3)	4 (3.1)
COPD	375 (1.3)	178 (2.1)	191 (2.1)	80 (2.6)	7 (5.3)
Renal disease	277 (1.0)	80 (0.9)	87 (1.0)	60 (2.0)	4 (3.1)
Peripheral vascular disease	262 (0.9)	97 (1.1)	103 (1.1)	60 (2.0)	5 (3.8)
Exposure to injectable GC, *n* (%)	9398 (32.6)	3025 (35.0)	3152 (35.0)	1214 (39.7)	49 (37.4)
Exposure to oral GC, *n* (%)	12,240 (42.4)	8,475 (98.2)	8,851 (98.2)	3,021 (98.9)	129 (98.5)
Exposure to nonbiologic DMARD, *n* (%)	22,097 (76.5)	7372 (85.4)	7701 (85.5)	2586 (84.6)	103 (78.6)
Exposure to biologic DMARD, *n* (%)					
Adalimumab	9636 (33.4)	3086 (35.8)	3213 (35.7)	1066 (34.9)	48 (36.6)
Certolizumab pegol	776 (2.7)	213 (2.5)	224 (2.5)	71 (2.3)	3 (2.3)
Etanercept	13,052 (45.2)	3842 (44.5)	4009 (44.5)	1366 (44.7)	55 (42.0)
Golimumab	781 (2.7)	212 (2.5)	224 (2.5)	75 (2.5)	5 (3.8)
Infliximab	4615 (16.0)	1279 (14.8)	1341 (14.9)	477 (15.6)	20 (15.3)
HIEs, *n* (%)	276 (1.0)	111 (1.3)	116 (1.3)	53 (1.7)	1 (0.8)

Dose cohorts for demographic and clinical descriptions are based on status at index date and are mutually exclusive

*COPD* chronic obstructive pulmonary disease, *DMARD* disease-modifying antirheumatic drug, *GC* glucocorticoid, *HIEs* hospitalized infectious events

^a^Very low dose GC cohort is a subset of the low-dose GC cohort

^b^Very high dose GC cohort is a subset of the high-dose GC cohort

## IRs of HIEs

**Fig. 1:**
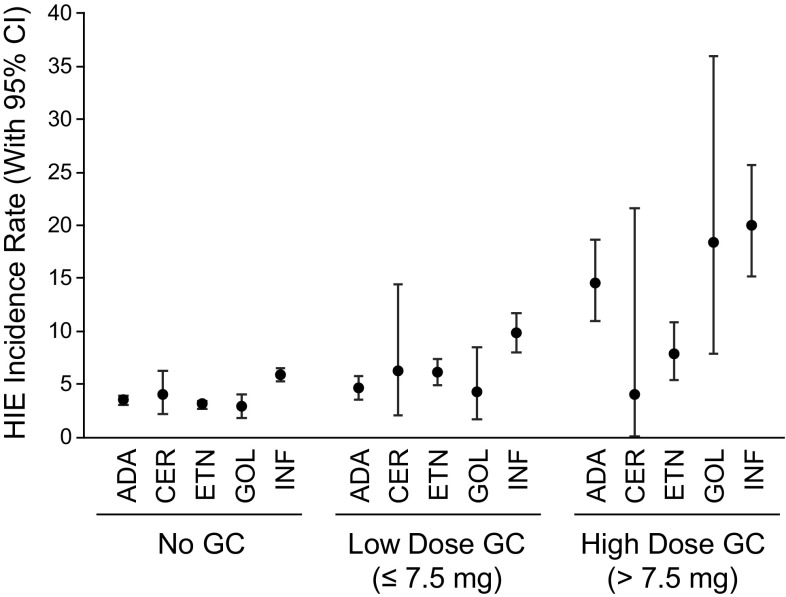
Incidence rates of HIEs stratified by index TNFi medication. HIE incidence rates are shown for patients receiving TNFi medication exposed to no GC, low-dose GC, or high-dose GC.* Error bars* represent 95% CI. *ADA* adalimumab, *CER* certolizumab pegol, *CI* confidence interval, *ETN* etanercept, *GC* glucocorticoid, *GOL* golimumab, *HIE* hospitalized infectious event, *INF* infliximab, *TNFi* tumor necrosis factor inhibitor

HIE IRs were similar for all patients receiving low-dose GC (≤7.5 mg) and the subset of patients with very low-dose GC (≤5.0 mg), and increased with increasing GC dose (Table 2). IRs were higher for patients ≥65 years of age in the no GC cohort and across all GC dose cohorts. A post hoc analysis of HIEs was conducted for the subset of patients receiving a very low GC dose (<5 mg) compared with patients who received exactly 5 mg. These results were consistent with the trend showing decreased incidence of HIEs with decreasing dose: the IR per 100 patient-years (95% CI) was 5.7 (4.23–7.43) for patients who received <5 mg GC, and 6.7 (5.74–7.48) for patients who received exactly 5 mg GC. A sensitivity analysis using a cut-off of >10 mg based on the ACR definition of high dose was also consistent with the GC dose relationship to HIEs: the IR per 100 patient-years (95% CI) was 26.4 (20.40–32.33) for patients who received >10 mg GC. For patients aged <65 years and ≥65 years, respectively, the IRs (95% CI) were 22.5 (16.36–28.57) and 43.4 (25.67–61.16) for GC >10 mg. There was a trend toward lower IRs with longer duration of GC exposure across all GC dose cohorts (Table 3). IRs for HIEs were generally similar across TNFi medications for patients receiving no GC and low-dose GC, although infliximab appeared to have the highest IRs for all GC dose cohorts (Fig. 1). For patients receiving high-dose GC, results were variable among the TNFi medications, possibly due to the relatively small number of patient-years of exposure for this GC dose cohort. The most common infections requiring hospitalization across all GC dose cohorts were pneumonia, cellulitis/abscess, and septicemia (Table 4).

**Table 2:** Summary of IRs of HIEs stratified by age group

	No GC	Very low dose GC^a^ (≤5.0 mg)	Low-dose GC (≤7.5 mg)	High-dose GC (>7.5 mg)	Very high dose GC^b^ (>20.0 mg)
All ages					
Total patient-years	23,654.2	4375.4	4603.1	1211.6	45.0
IR per 100 patient-years	3.9	6.4	6.4	13.3	24.5
(95% CI)	(3.63–4.13)	(5.65–7.17)	(5.68–7.16)	(11.32–15.51)	(12.21–43.76)
Ages <65 years					
Total patient-years	20,085.2	3463.1	3634.0	995.2	35.8
IR per 100 patient-years	3.2	4.7	4.7	11.7	22.3
(95% CI)	(2.91–3.40)	(4.04–5.52)	(4.00–5.44)	(9.63–13.98)	(9.64–43.98)
Ages ≥65 years					
Total patient-years	3,569.0	912.3	969.1	216.4	9.1
IR per 100 patient-years	8.0	12.6	12.8	20.8	32.9
(95% CI)	(7.06–8.94)	(10.41–15.13)	(10.64–15.26)	(15.17–27.83)	(6.77–95.99)

*CI* confidence interval, *GC* glucocorticoid, *HIE* hospitalized infectious event, *IR* incidence rate

^a^Very low dose GC cohort is a subset of the low-dose GC cohort

^b^Very high dose GC cohort is a subset of the high-dose GC cohort

**Table 3:** Summary of IRs of HIEs stratified by follow-up time

	1–14 days follow-up	15–29 days follow-up	30–59 days follow-up	≥60 daysfollow-up
No GC				
No. of HIE cases (total patient-years)	54 (1,494.2)	76 (1515.7)	122 (2694.5)	664 (17,949.8)
IR per 100 patient-years (95% CI)	3.6 (2.71–4.72)	5.0 (3.95–6.28)	4.5 (3.76–5.41)	3.7 (3.42–3.99)
Rate ratio (95% CI)	Reference	1.4 (0.98–1.97)	1.3 (0.91–1.73)	1.0 (0.78–1.35)
Low-dose GC				
No. of HIE cases (Total patient-years)	42 (625)	34 (549.9)	62 (790.2)	156 (2,638.1)
IR per 100 patient-years (95% CI)	6.7 (4.84–9.08)	6.2 (4.28–8.64)	7.9 (6.02–10.06)	5.9 (5.02–6.92)
Rate ratio (95% CI)	Reference	0.9 (0.59–1.45)	1.2 (0.79–1.73)	0.88 (0.63–1.24)
High-dose GC				
No. of HIE cases (total patient-years)	47 (356.5)	23 (230.3)	34 (246.0)	57 (378.8)
IR per 100 patient-years (95% CI)	13.2 (9.69–17.53)	10.0 (6.33–14.99)	13.8 (9.57–19.32)	15.1 (11.4–19.5)
Rate ratio (95% CI)	Reference	0.8 (0.46–1.25)	1.1 (0.67–1.63)	1.1 (0.78–1.68)

*CI* confidence interval, *GC* glucocorticoid, *HIE* hospitalized infectious event, *IR* incidence rate

**Table 4:** Most common infections requiring hospitalization

Infection,* n* patients (% of HIEs)	No GC (916 HIEs)	Very low dose GC (279 HIEs)	Low-dose GC (294 HIEs)	High-dose GC (161 HIEs)	Very high dose GC (11 HIEs)
Pneumonia (organism unspecified)	181 (19.8)	59 (21.2)	64 (21.8)	31 (19.3)	3 (27.3)
Cellulitis/abscess	101 (11.0)	31 (11.1)	31 (10.5)	16 (9.9)	1 (9.0)
Septicemia	92 (10.0)	37 (13.3)	38 (12.9)	19 (11.8)	1 (9.0)
Pleurisy	69 (7.5)	17 (6.1)	17 (5.8)		
Disorders of urethra/urinary tract	63 (6.9)	18 (6.5)	22 (7.5)	10 (6.2)	1 (9.0)

*HIEs* hospitalized infectious events, *GC* glucocorticoid, *HIEs* hospitalized infectious events

### Adjusted rate ratios for HIEs

After controlling for variables of baseline GC dose, age, use of injectable or oral GCs, use of nonbiologic DMARDs, comorbidities (diabetes, asthma, renal disease, congestive heart failure, hypertension, peripheral vascular disease, and chronic obstructive pulmonary disease (COPD); assessed individually), baseline HIEs, and exposure to TNFi medications, the adjusted rate ratio (95% CI) for low-dose GC vs no GC was 1.4 (1.19–1.58), for high-dose GC vs no GC was 2.8 (2.30–3.31), and for high-dose GC vs low-dose GC was 2.0 (1.65–2.44) (Table 5). These variables were also independent predictors of HIE risk for both low- and high-dose GCs except for peripheral vascular disease, which was a predictor of HIE risk for only high-dose GCs. Infliximab was independently associated with a higher rate of infection among all steroid dose comparisons, while adalimumab was associated with a slight increase in the high-dose to no GC comparison.

**Table 5:** Adjusted rate ratios for HIEs for no GC, low-dose GC, and high-dose GC

	Low-dose GC vs No GC		High-dose GC vs No GC		High-dose vs Low-dose GC	
	RR	95% CI	RR	95% CI	RR	95% CI
Crude rate ratios	1.7	1.45–1.88	3.4	2.90–4.06	2.2	1.78–2.61
Adjusted rate ratios	1.4^‡^	1.19–1.58	2.8^‡^	2.90–4.06	2.0^‡^	1.65–2.44
Age (continuous)	1.0^‡^	0.97–0.98	1.0^‡^	0.97–0.98	1.0^‡^	0.96–0.98
Exposure to injectable GC	1.3^‡^	1.17–1.47	1.3^‡^	1.16–1.48	1.3*	1.07–1.56
Exposure to oral GC	1.2*	1.09–1.42	1.2*	1.07–1.40	0.8	0.56–1.08
Exposure to nonbiologic DMARD	0.7^‡^	0.64–0.86	0.8*	0.67–0.91	0.7*	0.53–0.86
Diabetes	1.5^‡^	1.24–1.82	1.7^‡^	1.37–2.01	1.1	0.82–1.58
Asthma	2.0^‡^	1.42–2.73	1.8^†^	1.29–2.57	1.1	0.65–2.04
Renal disease	1.7*	1.21–2.41	1.7*	1.22–2.46	1.4	0.86–2.37
Congestive heart failure	1.4*	1.08–1.83	1.4*	1.08–1.88	1.3	0.88–1.94
Hypertension	1.4^†^	1.16–1.62	1.3*	1.07–1.52	1.5^†^	1.18–1.93
Peripheral vascular disease	1.2	0.82–1.81	1.4	0.97–2.14	0.5*	0.22–0.93
COPD	2.0^‡^	1.53–2.73	2.0^‡^	1.44–2.69	2.2^‡^	1.49–3.34
HIE during baseline	1.8^†^	1.28–2.58	1.6*	1.11–2.44	2.0*	1.22–3.39
Etanercept	Reference		Reference		Reference	
Adalimumab	1.1	0.91–1.23	1.3*	1.07–1.47	1.1	0.84–1.36
Infliximab	1.6^‡^	1.38–1.84	1.8^‡^	1.50–2.05	1.6^‡^	1.29–2.06
Golimumab	0.9	0.62–1.28	1.1	0.77–1.59	1.2	0.71–2.10
Certolizumab pegol	1.1	0.73–1.74	1.1	0.69–1.81	0.8	0.37–1.92

*RR* rate ratio, *GC* glucocorticoid, *DMARD* disease-modifying antirheumatic drug, *COPD* chronic obstructive pulmonary disease, *CI* confidence interval

*P ≤ 0.05

^†^ *P* ≤ 0.001

^‡^ *P* ≤ 0.0001

## Discussion

Rates of HIEs in this analysis of patients with RA were lowest for patients on no GC and increased with increasing dose of oral GCs. In a similar analysis of RA patients, the overall rate of infections requiring hospitalization was 9.6 cases per 100 person-years [1], compared with IRs of 3.9, 6.4 and 13.3 per 100 patient-years for patients on no GC, low-dose GC, and high-dose GC, respectively, in our study. Rates of HIEs were fairly similar across TNFi medications for each GC dose cohort; although patients on infliximab had consistently higher rates of HIEs in each GC dose cohort. These results are consistent with an analysis of data from the US Veterans Administration, in which rates of hospitalizations for bacterial infections were similar between adalimumab, and etanercept, and higher for infliximab [7]. Results from that study also showed that patients receiving doses of prednisone >7.5 mg/day were at increased risk of infection [7]. Data from the Canadian BioTRAC registry showed that RA patients treated with infliximab receiving GCs at a dose >5 mg were 2.48 times more likely to develop an infection than patients with no GC exposure [11].

The trend of GC-dose-dependent increased rates of infection has been reported previously. Dixon et al. showed that increases in GC dose led to increases in relative risk for infection, with relative risks of 2.5, 3.0, and 4.3 for GC doses of 5, 10, and >20 mg, respectively [12]. RA therapies and treatment status (naïve or previously treated) were not reported, and the authors noted that adjustment for comorbidity and RA therapies led to estimates that were ~40% higher than the unadjusted estimates. Data from the German biologics register RABBIT showed that the IR ratios (95% CI) of infections in patients receiving a very high GC dose (≥15 mg) or a high GC dose (7.5–14 mg) were 4.7 (2.4–9.4) and 2.1 (1.4–3.2), respectively, compared to patients with no GC exposure [13].

The ACR defines low-dose and high-dose GCs as ≤10 mg and >10 mg, respectively [10]. Based on this cut-off, we conducted a sensitivity analysis and found that the IR (95% CI) for HIEs was 26.4 (20.4–32.33) per 100 patient-years for all patients receiving>10 mg and a notable 43.4 (25.67–61.16) for patients ≥65 years of age. These results are consistent with findings from a study that was designed to identify risk factors for serious infections in RA patients. In that study, patients receiving a dose >10 mg were 3.97 times more likely to develop a serious infection compared to patients on no GC, and that patients aged ≥80 years were at higher risk for developing an infection than patients aged <80 years (HR 2.18; 95% CI 1.21–3.91) [14]. The high risk of infection needs to be considered when selecting a GC dose for elderly patients with RA.

Per the new ACR guidelines for the treatment of RA, combination therapy can be steroid sparing [10]. Our analysis showed that exposure to nonbiologic DMARDs during the baseline period was associated with a 20–30% reduction in HIE rate ratios. This finding could be due to better disease control with combination therapy or channeling of patients at high risk of infections away from combination therapy. Our analyses were not stratified by concomitant use of nonbiologic DMARD therapies throughout the follow-up period, and further research is required to address the impact of combination therapy on HIE rates.

Age was a strong predictor of HIE risk for both low and high GC doses. Additionally, comorbidities of diabetes, asthma, renal disease, congestive heart failure, hypertension, and COPD were strong predictors of HIE risk for low and high GC doses. Hypertension, peripheral vascular disease, and COPD were predictors of HIE risk for high-dose GC but not for low-dose GC. Among the individual TNFi treatments, infliximab was associated with a higher rate of infection at both the low- and high-dose comparisons, consistent with other studies [8, 15]. Notably, the infliximab cohort was older than the other TNFi cohorts (data not shown), and a high proportion of patients ≥65 years of age may have also contributed to the higher HIE rate for infliximab. This observation may be due in part to healthcare coverage for elderly patients: infused drugs such as infliximab are covered by Medicare Part B coverage whereas self-injected TNFi medications are not. Sample size was too small to allow for analyses by individual TNFi medications among those patients >65 years of age.

This study was unique in that it included the assessment of HIEs in patients newly initiating TNFi therapy, and also considered the duration of both TNFi and GC exposures and GC dose levels. A strength of the study was the amount of data available from the commercial claims database. The large patient population allowed for the evaluation of different dose levels and GC dose categories. The claims database also provides information on many variables (demographic, diagnostic, prescription claims for concurrent medications, etc.) that contributed to the analysis. The study also had some limitations. While underlying disease activity was not known, we did limit the analysis to patients who were newly initiating TNFi therapy and therefore expected to be comparable with relation to disease activity. Unmeasured confounding of disease severity is therefore a limitation of this study. TNFi exposure and oral GC dose levels were defined using administrative claims and pharmacy data, resulting in an inherent risk of misclassification error. Prednisone tapering may have occurred; however, based on limitations of the dataset, we were unable to identify when this occurred. Outpatient prescription claims used to identify therapies only indicated that a prescription was filled, but provided no information on actual utilization of the medication by the patient. A patient might not submit a reimbursement claim for a pharmacy-filled GC prescription, or might split doses to extend a prescription or to save cost; therefore, there is potential for misclassification of oral GC exposure status. The sample size was quite small for some subset analyses, particularly in the high-dose category, and the results of those analyses should be interpreted with caution.

In conclusion, doses of oral GCs as low as 5 mg were associated with an increased risk of infection in this cohort of RA patients. As with other subsets of the RA population, clinicians need to consider the steroid dose when evaluating the risk of infection among patients newly initiating TNFi therapies.
